# Loss of Complement Factor H impairs antioxidant capacity and energy metabolism of human RPE cells

**DOI:** 10.1038/s41598-020-67292-z

**Published:** 2020-06-25

**Authors:** Angela Armento, Sabina Honisch, Vasiliki Panagiotakopoulou, Inga Sonntag, Anke Jacob, Sylvia Bolz, Ellen Kilger, Michela Deleidi, Simon Clark, Marius Ueffing

**Affiliations:** 1 Institute for Ophthalmic Research, Department for Ophthalmology, Tübingen, Germany; 2 German Center for Neurodegenerative Diseases (DZNE), Tübingen, Germany; 3 Hertie-Institute for Clinical Brain Research, University of Tübingen, Tübingen, Germany

**Keywords:** Retina, Stress signalling

## Abstract

Polymorphisms in the Complement Factor H (*CFH*) gene, coding for the Factor H protein (FH), can increase the risk for age-related macular degeneration (AMD). AMD-associated *CFH* risk variants, Y402H in particular, impair FH function leading to complement overactivation. Whether this alone suffices to trigger AMD pathogenesis remains unclear. In AMD, retinal homeostasis is compromised due to the dysfunction of retinal pigment epithelium (RPE) cells. To investigate the impact of endogenous FH loss on RPE cell balance, we silenced *CFH* in human hTERT-RPE1 cells. FH reduction led to accumulation of C3, at both RNA and protein level and increased RPE vulnerability toward oxidative stress. Mild hydrogen-peroxide exposure in combination with *CFH* knock-down led to a reduction of glycolysis and mitochondrial respiration, paralleled by an increase in lipid peroxidation, which is a key aspect of AMD pathogenesis. In parallel, cell viability was decreased. The perturbations of energy metabolism were accompanied by transcriptional deregulation of several glucose metabolism genes as well as genes modulating mitochondrial stability. Our data suggest that endogenously produced FH contributes to transcriptional and metabolic homeostasis and protects RPE cells from oxidative stress, highlighting a novel role of FH in AMD pathogenesis.

## Introduction

Retinal pigment epithelium (RPE) cells are crucial for the maintenance of retinal homeostasis. RPE cells are located on a thin membrane called Bruch´s membrane (BM), and together provide a barrier between the neuroretina and the choroid capillary network. In addition, RPE cells fulfil several key functions, such as phagocytosis of the photoreceptor outer segments, transport of nutrients, preservation of the retinal structure and, most importantly, due to their high antioxidant capacity, RPE cells protect the retina from photo-oxidation and oxidative damage^1^. RPE dysfunction and degeneration are key features of age-related macular degeneration (AMD), a complex degenerative disease, and the primary cause of blindness in the elderly population^2^. AMD is characterized by a progressive degeneration of the macula, the cone-rich area of the retina, where damage in this area leads to central vision loss and ultimately blindness^3^. The aetiology of AMD involves ageing processes, genetic predisposition and environmental factors, however a full understanding of AMD pathogenesis is lacking, which makes drug discovery challenging^4^. A defining hallmark of AMD is the presence of deposits, called drusen, between the BM and the RPE layer^5^. In the presence of drusen or altered extracellular matrix (ECM) of BM, the functionality of RPE cells may be impaired^6^. The retinal microenvironment is already highly oxidized in physiological conditions, due to a very high energy demand and photo-oxidation. Ageing processes, in combination with external stressors including smoking or a high fat diet^7,8^, force RPE cells to deal with excessive levels of oxidative stress. Disturbed RPE cell homeostasis, and in particular RPE cell metabolism, has been recently introduced as an important aspect of AMD pathology^9^. In fact, the energy metabolism of primary RPE cells isolated from AMD patients was found to be strongly impaired compared to RPE cells from healthy controls^10^. In this model, glycolysis as well as mitochondrial respiration were reduced in RPE cells from AMD patients^10^. Other studies showed that mitochondrial dysfunction in RPE cells may represent a relevant AMD feature. Of the proteins differentially expressed in RPE cells from AMD donors and healthy controls, many are mitochondrial proteins^11^. Moreover, it has been shown in a mouse model that conditionally-induced mitochondrial damage in RPE cells leads to cell metabolic reprogramming and photoreceptors malfunction^12^.

So far, there is lack of knowledge on the impact of the high-risk genetic variants on RPE cell homeostasis. A large portion of AMD genetic high-risk variants is located in genes coding for complement system regulatory proteins (FH, FI, C3, FB/C2)^13^. In particular, a common polymorphism in the complement factor H gene (*CFH*), leads to an amino acid exchange from a tyrosine to histidine at position 402 (Y402H) in the two proteins encoded by the gene: factor H (FH) and its truncated splice variant factor H-like protein 1 (FHL-1)^14^. This common polymorphism is strongly associated with an increased risk for AMD and may account for ~50% of AMD cases in the United States^15^. The mechanism by which the FH H402 variant confers predisposition for AMD is not clear. The AMD-associated FH H402 variant impairs FH and FHL-1 function, leading to uncontrolled complement system activation *in vitro*^16^ and reduced binding affinity to heparan sulphate chains in the BM^17^. Recent studies highlighted the possibility that FH is not only contributing to AMD through its classical role in complement regulation, but may have a non-canonical role, influencing other processes. For example, it has been shown that the FH H402 variant presents altered binding affinity to C-reactive protein, indicating a possible role in the inflammatory response^18^. FH has also been associated with lipid metabolism; a process which is also altered in AMD^7^. Indeed, FH H402 shows an impaired binding to malondialdehyde, product of lipid oxidation^19^, and aged mice carrying FH H402 variant presented altered lipoprotein levels^20^. In addition, exogenously applied FH protects RPE cells from oxidized lipid-mediated damage^21^. The non-canonical role of the complement system has also been investigated in other cell types and diseases. For example in T cells, local C3b accumulation drives the complement receptor CD46-mediated metabolic changes that are necessary for Th1 cell activation upon T cell receptor (TCR) stimulation^22^. On the other hand, C3 can be cleaved to C3b and C3a intracellularly by cathepsin L (CTSL), ensuring basal metabolic activity in the resting state of T cells^23^. Dysregulation of this process leads to T cell activation in the joints of patients with juvenile idiopathic arthritis (JIA)^23^. FH can also be internalized by apoptotic cells (T cells and ARPE19 cells) to mediate CTSL-mediated cleavage of C3 and improve the clearance of apoptotic cells^24^.

This study investigates the impact of endogenous FH loss on RPE cell metabolism and their subsequent vulnerability toward oxidative stress. We employed RNA interference to decrease FH levels in the hTERT-RPE1 human cell line. In FH deprived RPE cells we observed an increase in C3 RNA and protein levels. Using the Seahorse Extracellular Flux Analyzer to measure bioenergetics, we observed that knock-down of the *CFH* gene negatively affects mitochondrial and glycolytic function of RPE cells when compared to controls. This impairment was even more pronounced when cells were exposed to oxidative stress by pre-treatment with hydrogen peroxide. The changes in energy metabolism were paralleled by transcriptional regulation of glucose metabolism and mitochondria stability genes. RPE cells lacking FH and exposed to the oxidative insult showed an increase in lipid peroxidation and a decrease in cell viability. Our results suggest that endogenous FH, produced by RPE cells, not only modulates the extracellular microenvironment *via* its regulation of C3 levels, but also has an intracellular impact on the antioxidant functions and metabolic homeostasis of RPE cells.

## Results

### FH reduction leads to extracellular C3/C3b accumulation

**Figure 1:**
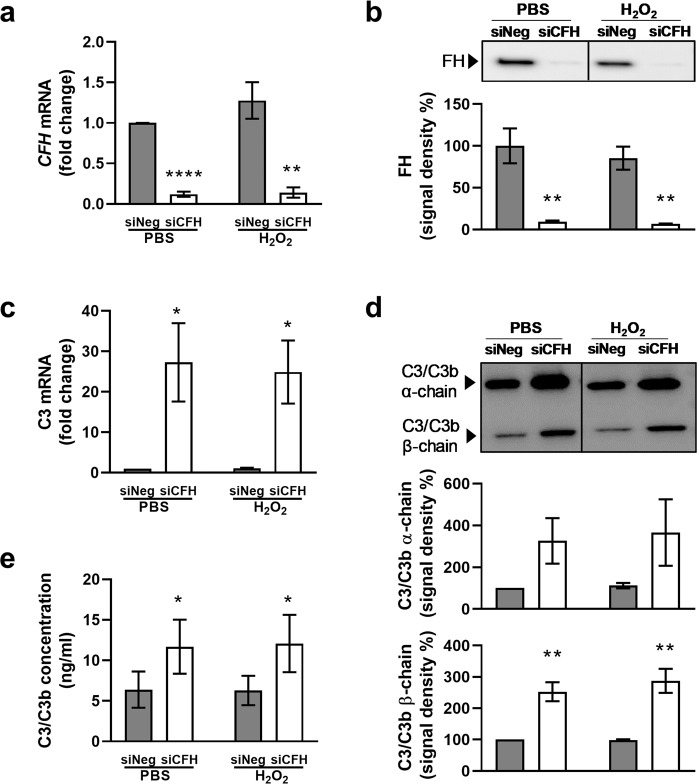
FH reduction leads to extracellular C3/C3b accumulation. hTERT-RPE1 cells were seeded, left to attach overnight and silenced for 24 hours with negative control (siNeg) or *CFH* specific (si*CFH*) siRNA. Cells were exposed for 90 minutes to 200 µM H_2_O_2_ or PBS and cell pellets and cell culture supernatants were collected for further processing after 48 hours. (**a)** Monitoring of *CFH* expression by qRT-PCR analyses in silencing negative control (siNeg) and specific *CFH* silenced (si*CFH*) hTERT-RPE1 cells. Data are normalized to the housekeeping gene PRPL0 using Δ ΔCt methods. SEM is shown, n = 3. (**b)** Western blot analyses of FH protein levels in cell culture supernatants of hTERT-RPE1 in the same conditions as (**a)**. Quantification of signal density of 4 independent experiments is shown. (**c)** Monitoring of C3 expression by qRT-PCR analyses in silencing negative control (siNeg) and specific *CFH* silenced (si*CFH*) hTERT-RPE1 cells. Data are normalized to housekeeping gene PRPL0 using Δ ΔCt method. SEM is shown, n = 4. (**d)** Western blot analyses of C3 α-chain and β-chain protein levels in cell culture supernatants of hTERT-RPE1 cells. Quantification of signal density of 3 independent experiments is shown. (**e)** C3/C3b ELISA analyses of cell culture supernatants of hTERT-RPE1 cells. SEM is shown, n = 4. Western Blot images were cropped, and full-length blots are presented in Supplementary Fig. S5. Significance was assessed by Student’s t-test. *p < 0.05, **p < 0.01, *** p < 0.001, ****p < 0.0001.

AMD is a complex and slow progressing disease, where 2 or more factors need to co-exist to develop the disease. The set-up used in this work provides the chance to study *in vitro* the combination of two risk factors: endogenous FH dysregulation and oxidative stress. To investigate the role of FH, we used siRNA to silence the *CFH* gene in hTERT-RPE1 established cell lines and subsequently induced a mild oxidative stress through hydrogen peroxide pre-treatment (200 µM for 90 minutes). We monitored the efficiency of *CFH* silencing in all experimental conditions, including PBS and H_2_O_2_ pre-treated cells after 48 hours in culture. Significantly reduced *CFH* mRNA was detected in *CFH* knock-down cells compared to the siNeg control cells, achieving almost 90% silencing of the *CFH* gene (Fig. 1a). The FH protein was almost undetected in cell culture supernatants collected at the same time point from the si*CFH* cells compared to controls (Fig. 1b). The hTERT-RPE1 cells showed gene expression of RPE markers: Bestrophin 1 (BEST1) and Retinoid Isomerohydrolase (RPE65) (Supplementary Fig. S1a). Tight junction protein ZO-1 (TJP1) staining, while partially localized on the cell membrane, was speckled and not uniform (Supplementary Fig. S1b), as expected for not fully differentiated RPE cells. Depletion of the FH protein led to upregulation of the *C3* gene (Fig. 1c), followed by an increase in extracellular levels of C3: as observed by both Western blot and ELISA. C3 extracellular protein levels were found to be higher, as shown by the higher levels of C3 alpha and beta chains in si*CFH* cells (Fig. 1d). An ELISA that detects both C3 and C3b, cleaved product of C3 triggering the amplification of complement system activation^25^, revealed a 2-fold increase in detectable C3/C3b in cell culture media of *CFH*-knock-down cells (Fig. 1e). Formation of the terminal complement complex, assayed by a sC5b-9 ELISA, was undetectable in cell culture supernatants or lysates of hTERT-RPE1 cells in any of the experimental conditions (Supplementary Fig. S2).

### FH loss increases vulnerability toward oxidative stress in RPE cells

**Figure 2:**
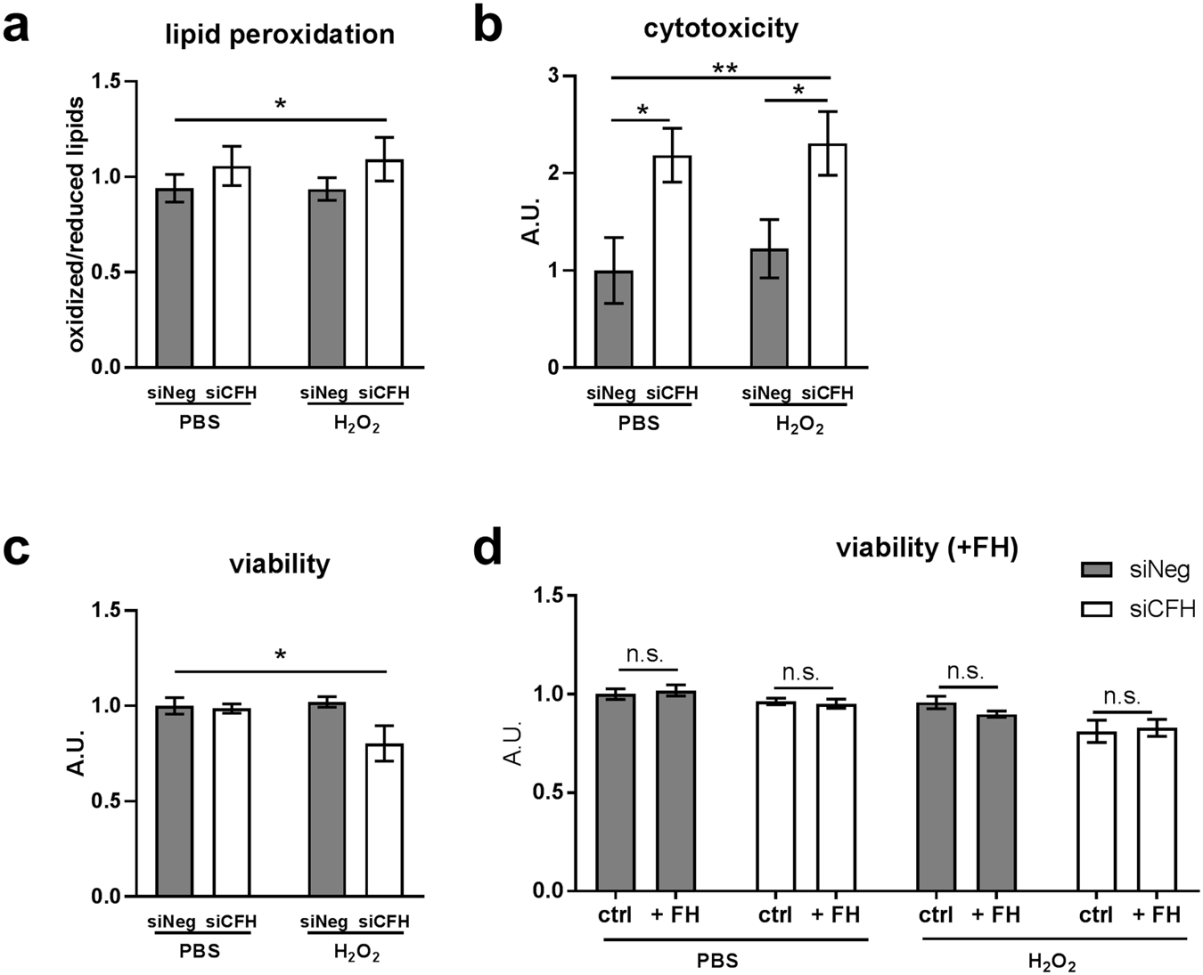
FH loss increases vulnerability of RPE cells toward oxidative stress. hTERT-RPE1 cells were seeded, left to attach overnight and silenced for 24 hours with negative control (siNeg) or *CFH* specific (si*CFH*) siRNA. Cells were exposed for 90 minutes to 200 µM H_2_O_2_ or PBS and specific dyes were added after 48 hours. (**a)** Lipid peroxidation levels were assessed *via* BODIPY® 581⁄591 C11 fluorescent dye. Fluorescence shift was measured at ~590 nm and ~510 nm. Data are shown as ratio oxidized/reduced lipids, higher bars indicate higher lipid peroxidation levels. SEM is shown, n = 7. (**b)** Cytotoxicity levels were assessed by cell-impermeable fluorescent dye bis-AAF-R110. SEM is shown, n = 5. (**c)** Viability was assessed by cell-permeable fluorescent dye GF-AFC (glycyl-phenylalanyl-aminofluorocoumarin). SEM is shown, n = 5. (**d)** Following addition of purified FH (1 µl/ml), viability was assessed by cell-permeable fluorescent dye GF-AFC (glycyl-phenylalanyl-aminofluorocoumarin). SEM is shown, n = 3. A.U. arbitrary units. Significance was assessed by Student’s t-test (single effect) and two-way ANOVA (combined effects) as described in the methods section. *p < 0.05, **p < 0.01.

In order to assess whether the silencing of *CFH* altered the response of hTERT-RPE1 cells to oxidative stress, we investigated cell lipid peroxidation levels after H_2_O_2_ treatment (Fig. 2a). In our model, lipid peroxidation levels were not affected by either FH deprivation or H_2_O_2_ pre-treatment alone. A small, but significant increase in lipid peroxidation levels was observed only in the absence of FH 48 hours after the oxidative treatment (Fig. 2a). As shown in Fig. 2c, cell viability was not affected in the absence of *CFH* expression in PBS alone, and pre-treatment with H_2_O_2_ had no effects on the siNeg control cells, confirming the known high antioxidant capacity of RPE cells^26^. However, cell viability was significantly reduced exclusively when RPE cells missing *CFH* expression were stimulated with H_2_O_2_ (Fig. 2c), indicating increased vulnerability toward a short exposure to oxidative stress in FH deprived RPE cells. Exogenously applied purified FH did not cause any significant change in the viability of hTERT-RPE1 cells deprived of FH, either in control conditions or after H_2_O_2_ exposure (Fig. 2d), highlighting the importance of endogenous FH in RPE cells. In parallel, we investigated cell membrane damage *via* a cytotoxicity assay. Silencing of *CFH* in RPE cells led to an increase in RPE cell damage, irrespective of H_2_O_2_-induced oxidative stress (Fig. 2b). In addition, FH deprivation led to alterations in ZO-1 staining (Supplementary Fig. S1b), as quantified by count and length of ZO-1 fragments (Supplementary Fig. S1c,d). Indeed, siNeg RPE cells showed a higher average number (109 vs 76, p < 0.0001) and average length (8.7 vs 7.9, p = 0.05) of ZO-1 linear fragments. This indicates that ZO-1 staining in siCFH RPE cells is more fragmented and areas of linear localization are less pronounced.

### FH loss impairs glycolysis in RPE cells

**Figure 3:**
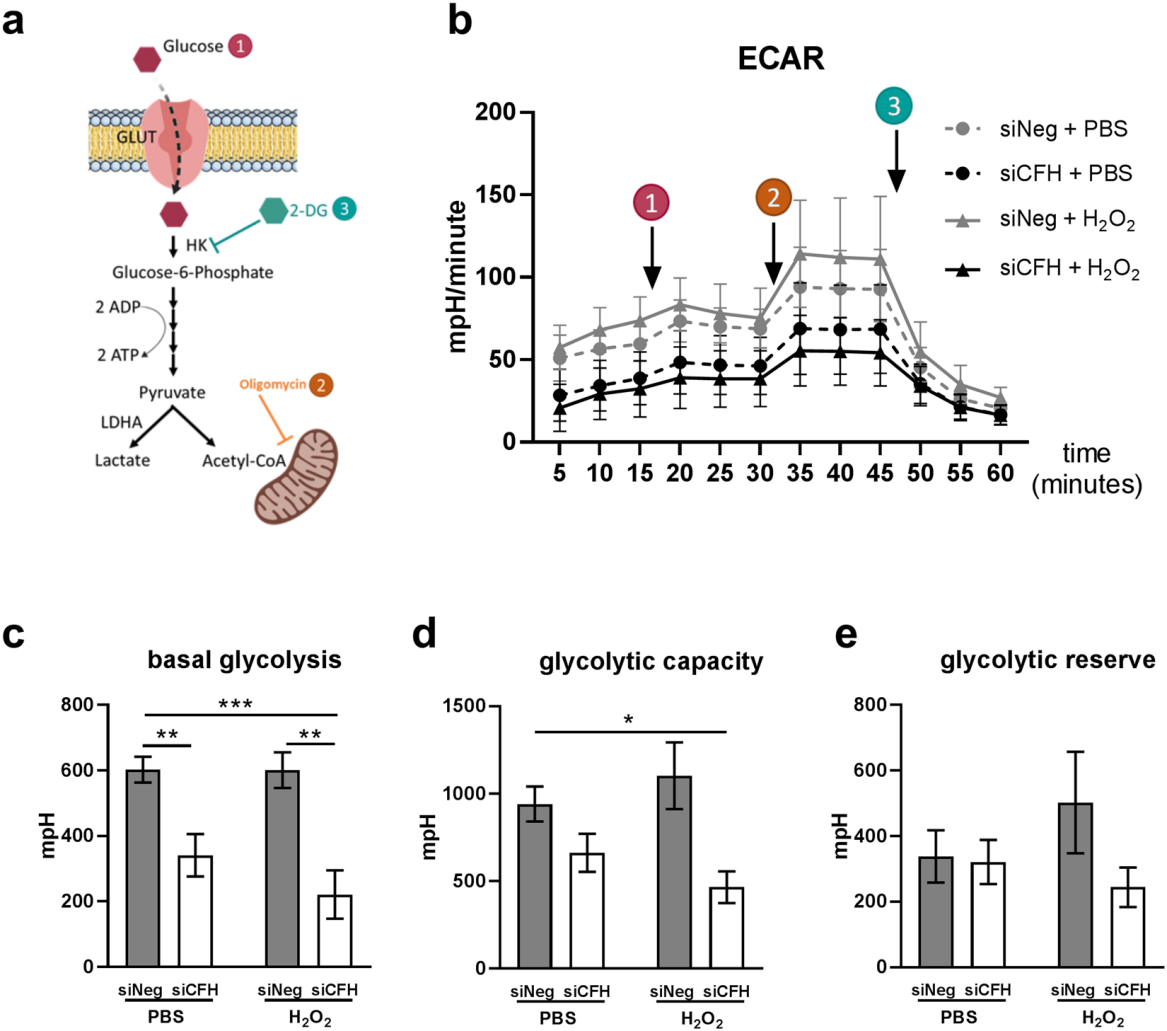
FH loss impairs glycolysis in RPE cells. (**a**) Schematic representation of glycolysis and steps targeted during Seahorse analyses (1,2,3). (**b)** hTERT-RPE1 cells were seeded, let attach overnight and silenced for 24 hours with negative control (siNeg) or *CFH* specific (si*CFH*) siRNA. 30,000 cells were transferred to Seahorse plates overnight and pre-treated for 90 minutes with 200 µM H_2_O_2_ or PBS. Curves show extracellular acidification rate (ECAR) measured after 48 hours. SEM is shown, n = 4–8. Arrows indicate injection of glucose (1), oligomycin (2) and 2-deoxyglucose (2-DG,3). (**c–e)** Parameters of glycolytic function are calculated from data shown in (**b**) and are expressed as total values for the 3 measurements (15 minutes). Basal glycolysis (**c**), glycolytic capacity (**d**) and glycolytic reserve (**e**). Significance was assessed by Student’s t-test (single effect) and two-way ANOVA (combined effects) as described in the methods section. *p < 0.05, **p < 0.01, ***p < 0.001.

**Figure 4:**
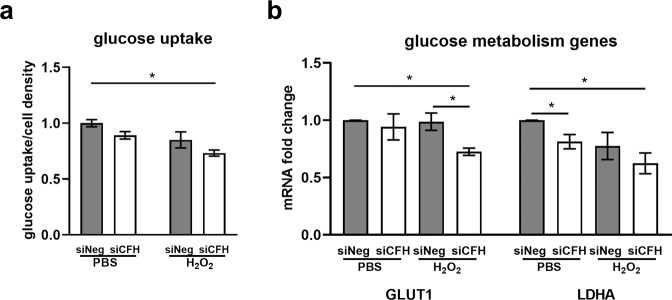
FH modulates glucose uptake and expression of glucose metabolism genes. hTERT-RPE1 cells were seeded, let attach overnight and silenced for 24 hours with negative control (siNeg) or *CFH* specific (si*CFH*) siRNA. Cells were exposed for 90 minutes to 200 µM H_2_O_2_ or PBS. **(a)** Glucose uptake was measured 48 hours after H_2_O_2_ pre-treatment in siNeg control cells and in si*CFH* cells. SEM is shown, n = 3. (**b)** gene expression analysis by qRT-PCR of glucose transporter 1 (GLUT1/SLC2A1) and glycolysis enzyme gene lactate dehydrogenase A (LDHA). SEM is shown, n = 3. Data are normalized to housekeeping gene PRPL0 using Δ ΔCt method. Significance was assessed by Student’s t-test (single effect) and two-way ANOVA (combined effects) as described in the methods section *p < 0.05.

To investigate the influence of FH on RPE cell metabolism, the extracellular acidification rate (ECAR) was monitored as an indication of glycolytic function using the glycolysis stress test. Figure 3a shows a schematic representation of glycolysis pathway highlighting the substrates and inhibitors used in the Seahorse analyses. Glucose, oligomycin and 2-deoxyglucose (2-DG) were sequentially injected (as shown by the arrows in Fig. 3b) to modulate glycolysis responses and ECAR. Figure 3b shows ECAR measurements in siNeg cells and si*CFH* cells pretreated with PBS or H_2_O_2_. Basal levels of glycolysis were found to be significantly lower by 43% in RPE cells deprived of FH, compared to siNeg controls (Fig. 3c). This reduction was even more pronounced when the *CFH* knock-down cells were pre-treated with H_2_O_2_ (Fig. 3c), with glycolysis being reduced by 63% compared to cells treated only with H_2_O_2_ (Fig. 3c). Glycolytic capacity was significantly reduced only when *CFH* knock-down cells were pre-treated with H_2_O_2_ (Fig. 3d), showing a reduction of 50% compared to siNeg control cells (Fig. 3d). Also, glycolytic reserve in si*CFH* H_2_O_2_ treated cells was slightly reduced compared to both siNeg controls (Fig. 3e). Consistently, glucose uptake was reduced significantly in si*CFH* cells after H_2_O_2_ exposure compared to siNeg control cells (Fig. 4a). In parallel, mRNA expression of the glucose transporter GLUT1 was reduced in H_2_O_2_-treated si*CFH* cells compared to both treated and untreated controls (Fig. 4b). Gene expression of LDHA (lactate dehydrogenase A), an isoform of LDH which preferentially converts pyruvate to lactate^27^, was also significantly reduced in all si*CFH* cells, more pronouncedly after peroxide treatment (Fig. 4b).

### FH loss impairs mitochondrial respiration in RPE cells

**Figure 5:**
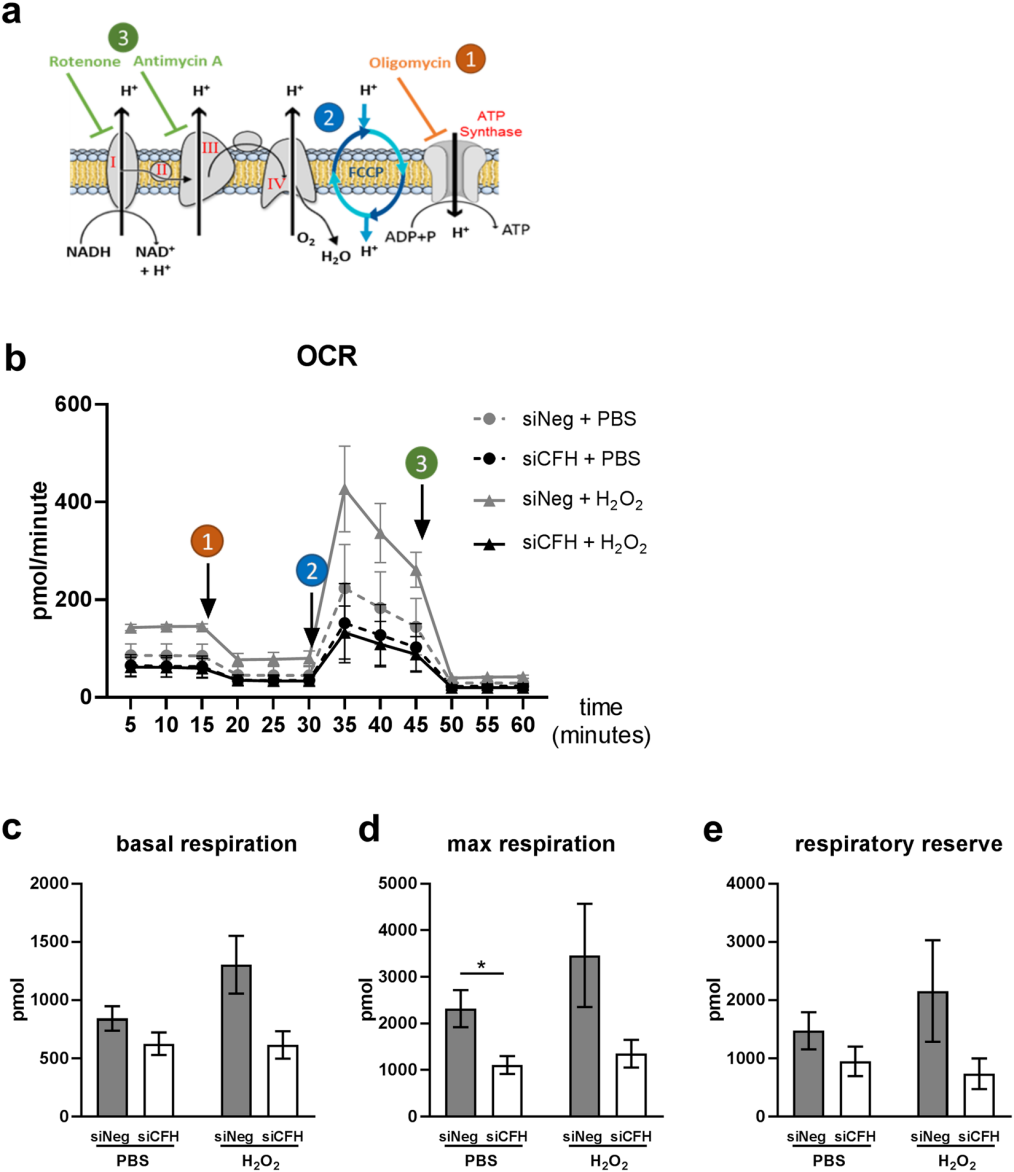
FH loss impairs mitochondrial respiration in RPE cells. (**a**) Schematic representation of oxidative phosphorylation chain and targeted steps during Seahorse analyses (1,2,3) (**b)** hTERT-RPE1 cells were seeded, let attach overnight and silenced for 24 hours with negative control (siNeg) or *CFH* specific (si*CFH*) siRNA. 30.000 cells were transferred to seahorse plates overnight and pre-treated for 90 minutes with 200 µM H_2_O_2_ or PBS. Curves show oxygen consumption rate (OCR) measured after 48 hours in hTERT-RPE1. SEM is shown, n = 4–8. Arrows indicate injection of oligomycin (1), FCCP (2) and antimycin and rotenone (3). (**c–e)** Parameters of mitochondrial function are calculated from data shown in (**b**) and are expressed as total values for the 3 measurements (15 minutes). Basal respiration (**c**), maximal respiration (**d**) and reserve respiratory capacity (**e**). Significance was assessed by Student’s t-test (single effect) and two-way ANOVA (combined effects) as described in the methods section *p < 0.05.

The potential influence of FH loss on mitochondrial respiration of RPE cells was assessed monitoring the oxygen consumption rate (OCR), an indication of mitochondria respiratory function using the cell mito stress test. Figure 5a shows a schematic representation of the oxidative phosphorylation (OxPhos) chain, highlighting the substances used in the Seahorse analyses. Oligomycin, carbonyl cyanide-4-(trifluoromethoxy)phenylhydrazone (FCCP) and antimycin/rotenone were sequentially injected (as shown by the arrows in Fig. 5b) to assess OCR in different conditions and calculate parameters of mitochondrial function. Figure 5b shows OCR measurements in siNeg cells and si*CFH* cells pretreated with PBS or 200 µM H_2_O_2_ for 90 minutes. All parameters of mitochondrial respiration showed a clear trend of reduction in all si*CFH* groups (Fig. 5c–e). However, the maximal respiration was significantly reduced in the absence of FH by 52% in PBS-treated cells (Fig. 5d). A slight increase in maximal respiration was observed when control cells were treated only with peroxide (Fig. 5d).

### FH loss alters the expression of mitophagy and mitochondria dynamics genes

**Figure 6:**
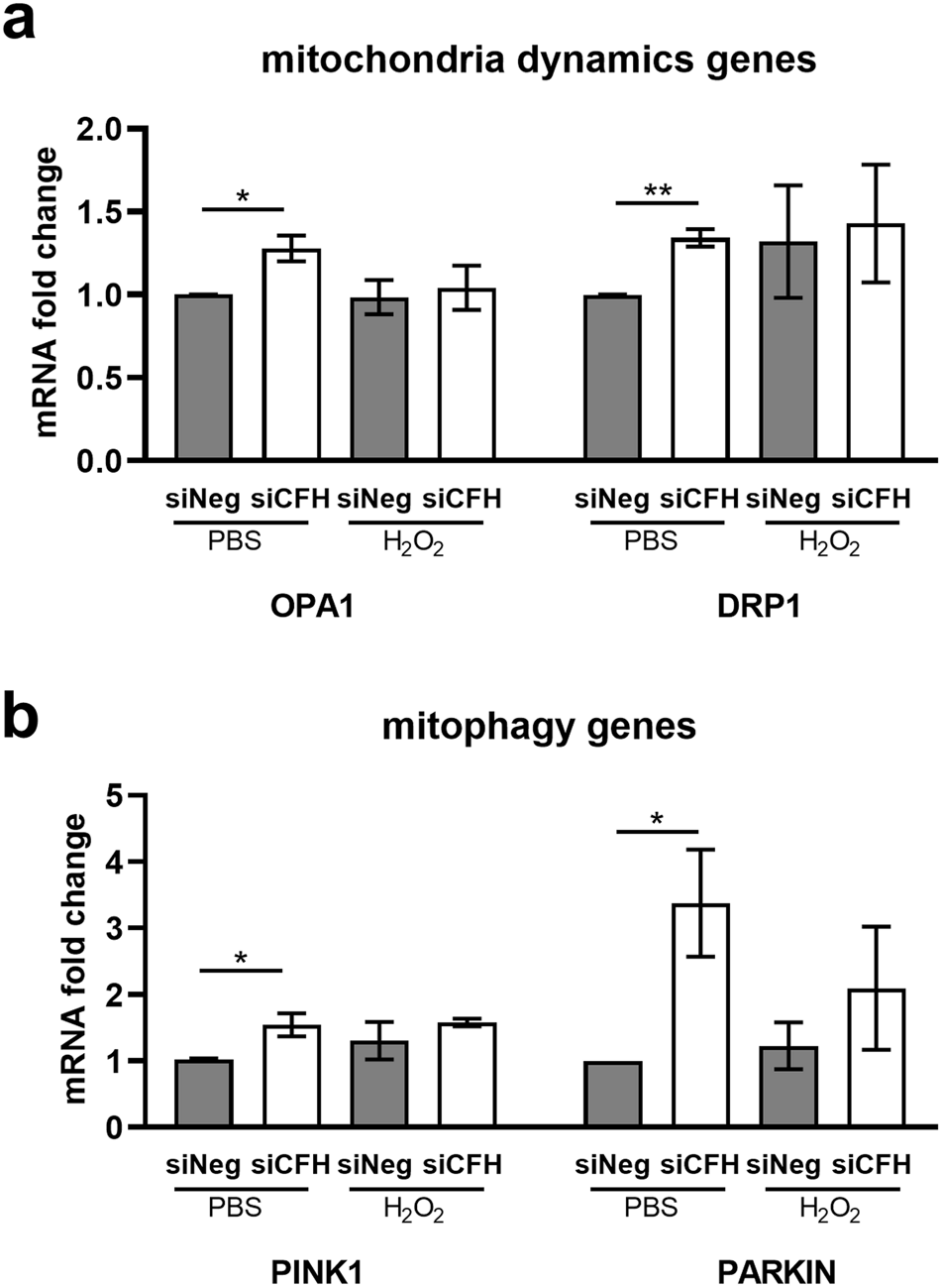
FH modulates expression of mitophagy and mitochondria dynamics genes. hTERT-RPE1 cells were seeded, left to attach overnight and silenced for 24 hours with negative control (siNeg) or *CFH* specific (si*CFH*) siRNA. Cells were exposed for 90 minutes to 200 µM H_2_O_2_ or PBS and RNA was collected after 48 hours. (**a)** gene expression analysis by qRT-PCR of genes involved in mitophagy processes: PTEN Induced Kinase 1 (PINK1) and E3 Ubiquitin-Protein Ligase Parkin (PARKIN) SEM is shown, n = 3. (**b)** Gene expression analysis by qRT-PCR of genes involved in mitochondria dynamics: OPA1 Mitochondrial Dynamin Like GTPase (OPA1) and Dynamin-Related Protein 1 (DRP1). SEM is shown, n = 3. Data are normalized to housekeeping gene PRPL0 using Δ ΔCt method. Significance was assessed by Student’s t-test *p < 0.05, **p < 0.01.

Several factors contribute to the energy metabolism regulation and antioxidant capacity of RPE cells, which often both rely on mitochondria function and stability^9,28^. Impairments in mitochondrial function can be caused by altered oxidative phosphorylation (OxPhos) chain components as shown for Alzheimer’s disease^29^, therefore we investigated by qPCR the expression of OxPhos genes NADH dehydrogenase 4 (ND4), Cytochrome c oxidase subunit 4 (COX4) and mitochondrial encoded ATP synthase 6 (ATP6) respectively components of complex 1, complex 4 and ATP synthase^30^ (shown in the schematic in Fig. 5a). No significant differences were observed in any of the experimental conditions (Supplementary Fig. S3a). Transcription factors promoting mitochondrial biogenesis like Peroxisome Proliferator-Activated Receptor Gamma Coactivator 1-Alpha (PGC1α) and Nuclear Factor, Erythroid 2 Like 2 (NRF2) have been shown to positively influence mitochondria metabolism and antioxidant response^31,32^. To test whether FH loss leads to a dysregulation of those factors, we analyzed gene expression levels of PGC1α and NRF2 (Supplementary Fig. S3b). No differences were detected for NRF2, where conversely PGC1α levels were higher in absence of FH. This result would suggest an improvement in mitochondria function, which was not the case in our model. We also measured transcriptional levels of antioxidant enzymes which are induced by PGC1α^33^, like Peroxiredoxin 3 (PRDX3), Catalase (CAT), Glutathione Peroxidase 1 (GPX1). We found no differences in PRDX3, a slight upregulation of CAT and a significant increase in GPX1 in the absence of FH (Supplementary Fig. S3c). These data suggest that RPE cells lacking FH are unsuccessfully trying to respond to an oxidative stress situation. Another mechanism of mitochondrial quality control is mitophagy, a mitochondria specific autophagy^34^. We found a significant alteration in the expression levels of genes regulating mitophagy (Fig. 6a), like PTEN induced putative kinase 1 (PINK1) and E3 Ubiquitin-Protein Ligase Parkin (PARKIN) and mitochondria dynamics, like Dynamin-Related Protein 1 (DRP1) and OPA1 Mitochondrial Dynamin Like GTPase (OPA1) when FH was missing (Fig. 6b). An alteration in mitophagy levels and mitochondria dynamics would lead to an increase in damaged mitochondria or altered mitochondria turnover.

## Discussion

The retina is a highly organized multi-layered tissue and, in the early stages of AMD, BM/RPE layer is the first affected. BM, composed by overlapping extracellular matrix sheets, together with RPE cells separates the retina from the choroid capillary network. RPE cells actively transport nutrients, as glucose, to the retina and eliminate waste material, like oxidized lipids, into the blood stream. Like every other tissue, the retina undergoes changes with age, including variations in collagen types^35^, loss in elastin^36^ and increase in metalloproteinases^37^. BM becomes rigid and thickened, where lipids begin to accumulate underneath the RPE cells^38^. Lipid deposits together with lipofuscin, melanin and complement proteins are the main constituents of drusen, the hallmark lesions associated with AMD pathology^5,39^. Moreover, another age-related change in the BM is the reduction in heparan sulphate, one of the main ligand by which FH anchors to the ECM^40^. These alterations in BM structure alter physiological conductivity and therefore impede a correct transport of oxygen or nutrients from the choroidal network to the RPE cells^41–44^, resulting in a condition of hypoxia^45^ and starvation. In addition, lifestyle habits, like smoking or maintaining a high-fat diet, both risk factors for AMD^7,46^, add oxidative stress to the retina^47,48^, and in particular to the mitochondria of RPE cells and photoreceptors^12^. With age, and more pronounced in AMD patients, mitochondrial damage is augmented^11,49^, thus leading to energy misbalance in the RPE cells. A bioenergetics crisis of RPE cells has been postulated to be at the basis of AMD pathology, especially in relation to the interplay between RPE cells and photoreceptors^9^. Since not the entire elderly population (or the population that smoke) is affected by AMD, other susceptibility factors have to be postulated for RPE cells in AMD patients. Genetic predisposition plays an essential role in AMD pathogenesis and carriers of high-risk variants may not be efficiently responding to ageing processes and oxidative stress. FH risk variants, in particular Y402H, lead to dysregulation of complement activation and hold differential binding properties to oxidized lipids^50,51^. In this study, we asked to which extent FH dysregulation may alter mechanisms relevant to AMD, like energy metabolism and response to oxidative stress. In the early stages of the disease oxidative stress is limited and can be handled by RPE cells. In addition, BM is still intact and systemic FH cannot cross choroid/BM interface due to its size^52^. Therefore, an interplay of genetics and natural age-related changes within the retina are likely to drive onset and progression of early AMD. In consequence, we investigated whether RPE cells with reduced FH activity are more vulnerable to a mild oxidative insult: conditions that may better represent the early stages of AMD. The *in vitro* system used in this study allowed us to investigate the effects of endogenous FH specifically on RPE microenvironment without the influence of systemic alterations present in blood. The classical role of FH is to dampen activation of the alternative complement pathway. FH acts as cofactor for the proteolytic activity of Complement Factor I (FI) and displaces Complement Factor B (FB) from C3b. Both activities reduce the levels of C3 turnover and complement activation^50^. We show here that RPE cells produce FH, as well as C3. FH downregulation leads to an increase in C3 mRNA levels, followed by accumulation of C3 in the extracellular compartment. In parallel, we detected a slight reduction of secreted FH after H_2_O_2_ pre-treatment, a phenomenon previously observed in H_2_O_2_-treated ARPE19 cells^53^. In the afore mentioned study, in which RPE cells were treated with 500 µM H_2_O_2_, FH accumulates intracellularly, while in a different study, exposure to a mild and short oxidative insult (150 µM for 90 minutes) does not result in intracellular accumulation^54^. It is possible that FH internalization depends on the levels of oxidative stress and, under higher oxidative conditions, FH is recruited inside apoptotic cells to help in their opsonization and removal, as shown by Martin *et al*.^24^ However, this process does not influence our results, considering that *CFH* silencing reduces both *CFH* gene transcription and production of FH protein and therefore no secreted FH exists to be taken back up into the cell.

Recent studies implicate FH in lipid metabolism. The AMD-risk-associated FH variant (H402) leads to a retinal damage similar to AMD and promotes other aspects of the disease, including the accumulation of lipoproteins in a murine model^20^. Moreover, two FH redox forms have been identified in the circulating blood of AMD patients and those forms hold dual functions^55^. Indeed, the reduced and oxidized forms of FH, as well as FH-Y402 and FH-H402, have different binding affinities to oxidized lipids, which accumulate in drusen^51,55^. Exogenous FH has been shown to protect ARPE19 cells against H_2_O_2_-induced stress^55^ and recently, also against exposure to oxidized lipids 4-HNE (4-hydroxy-2-nonenal)^21^. In our study, we show for the first time a protective role of endogenous *CFH*/FH against oxidative insult. RPE cells, lacking FH, display an accumulation of oxidized lipids in response to H_2_O_2_ pre-treatment. As a consequence, cell viability of RPE cells is affected only in this condition. Interestingly, either FH loss or H_2_O_2_ treatment alone, had no negative effects on viability or lipid peroxidation, confirming the importance of risk factor combinations in RPE cell degeneration during AMD pathology. Lipid oxidation also affects membrane permeability^56^ and exogenous FH helps maintain a ZO-1 localization in response to 4-HNE in ARPE19 cells^21^. In our model, we show that reduction of endogenous FH mediates cell membrane damage in RPE cells. Disruption of the integrity of cell membrane could be caused by the deposition of the complement membrane attack complex (MAC), composed of sC5b-9^57^, on cell surfaces, but this was not the case in our model. On the other hand, we observed more fragmented ZO-1 distribution when RPE cells were deprived of FH.

Reduction in antioxidant capacity and viability, as well as membrane damage^58^, could underlie a misbalance in energy metabolism of RPE cells. Several recent studies show altered bioenergetics being part of AMD pathology^9,10^. Mitochondria account for the majority of cell energy production, *via* the tricarboxylic acid (TCA) cycle, OxPhos or lipid breakdown. Nevertheless, mitochondrial metabolism and glycolysis work in a synergetic way, since by-products of glycolysis, like pyruvate, can fuel the TCA. A disruption of either mitochondria or glycolytic function can lead to a failure in the metabolic system. Loss of *CFH* has been associated with mitochondria impairment in retinal development in a *CFH* knock-out mouse model^59^ and patients carrying the *CFH* H402 high-risk variant present increased mitochondrial DNA damage^60^. Whether FH contributes to metabolic homeostasis of RPE cells has never been investigated. We show that FH loss alters energy metabolism and lead to a phenotype similar to the one observed in primary RPE cells derived from AMD patients^10^. RPE cells deprived of FH, barely responded to FCCP, an uncoupling reagent which disrupts proton gradients in the mitochondria. As a consequence, the parameter mainly affected in the absence of FH was the maximal respiration, assessed by adding FCCP. Since RPE cells are highly dependent on mitochondria for their metabolism, they would tend, upon metabolic stress, to increase their mitochondrial respiration^61^. Indeed, maximal respiration was increased in control cells after hydrogen peroxide treatment, indicating that RPE cells may respond to a short oxidative insult by increasing their respiration. Of note, this phenomenon was completely abolished in si*CFH* cells. These findings indicate that cells lose their capacity to meet extreme metabolic challenges and that mitochondria membrane potential could be already altered in absence of FH alone. However, when maximal respiratory capacity is damaged, cells would generally switch to glycolysis, a phenomenon known as the Warburg effect^62^. But, in our model, glycolysis was also severely affected (already at basal levels in RPE cells deprived of FH) leading to a total failure in the metabolic system of RPE cells. In these circumstances, RPE cells were unable to respond appropriately and survive after the stress stimuli. In line with reduced glycolysis we observed low LDHA and GLUT1 levels in FH deprived cells. RPE GLUT1 levels are particularly important for the preservation of the neuroretina. Indeed, in mice with a severe reduction of GLUT1 in RPE cells, glucose transport to the retina was severely hindered and led to photoreceptor cell death^63^. Accumulation of intra- and extracellular C3b has been shown to be involved in the metabolic reprogramming of T cells. These changes include an increase in glycolysis, oxidative phosphorylation (OXPHOS) and uptake of glucose and amino acids, mediated by upregulation also of GLUT1^22,23^. As in T cells, our data suggest that complement factors play an important role in the maintenance of metabolic homeostasis of RPE cells as well.

Similar to AMD primary RPE cells, we see in si*CFH* cells upregulation of genes involved in antioxidant response, like CAT and GPX1, as well as transcription factors involved in mitochondria stability and biogenesis, like PGC1α. These factors are indicators of a response to oxidative stress and mitochondria damage, but they are not the only ones contributing to define whether a cell will successfully escape from excessive oxidative stress. In fact, we do not observe any improvement in mitochondria function, either in PBS or H_2_O_2_ treated cells, contrarily to AMD primary RPE cells which show a greater resistance toward oxidative stress after 24 hours^10^. This phenomenon may depend on the experimental time. Indeed, another study testing the effect of H_2_O_2_ after 48 hours, a time point used in our model, showed more damage in AMD primary RPE cells after oxidative stress exposure^64^. Cells have developed alternative mechanisms to overcome mitochondrial dysfunction which are activated in case of damage. Mitophagy is a specific type of autophagy directed in eliminating unnecessary, damaged or malfunctioning mitochondria^34^ and the complement system, specifically the lectin pathway, has already been proposed to be involved in the homeostatic clearance of mitochondria^65^. Mitophagy is classically mediated by the PINK-PARKIN axes^66^, and both genes were upregulated in RPE cells when FH levels were reduced. PINK1 accumulates on the membrane of damaged mitochondria and its kinase activity is required for recruitment of PARKIN in order to mediate ubiquitination and organelle removal^66^. PINK1 and PARKIN are also involved in the transport to the lysosomes of mitochondria-derived vesicles (MDV). MDVs contain oxidized proteins which are removed from the mitochondrial matrix and their removal can denote a first effort to rescue mitochondria before engaging in mitophagy^67^. MDV trafficking has been implicated in Parkinson’s and Alzheimer’s disease through association of the vps35 protein, which is mutated in the diseases and involved in MDVs transport^68–70^. In AMD, vesicles accumulation, alterations in autophagy and lysosomes in RPE cells have been described^71,72^, but a role of MDVs in AMD pathology has not yet been considered. PINK1 interacts with proteins involved in mitochondria dynamics including DRP1 and OPA1. Both are fission and fusion genes^73^, and were seen upregulated in *CFH* knock-down cells, highlighting the possibility that mitochondrial structures and dynamics are compromised by FH reduction. OPA1 and DRP1 work in concert to maintain mitochondrial stability. Indeed, DRP1 loss-of-function alters OPA1 processing, thus affecting the organization of mitochondrial membranes^74^. Moreover OPA1 is also involved in mitochondrial contraction and inner membrane depolarization, leading to proton leak^75^. Thus, the loss of FH activity likely promotes destabilization of mitochondrial structure and function, followed by perturbation in mitochondrial energy metabolism, structural maintenance of mitochondria and an increase in mitophagy.

**Figure 7:**
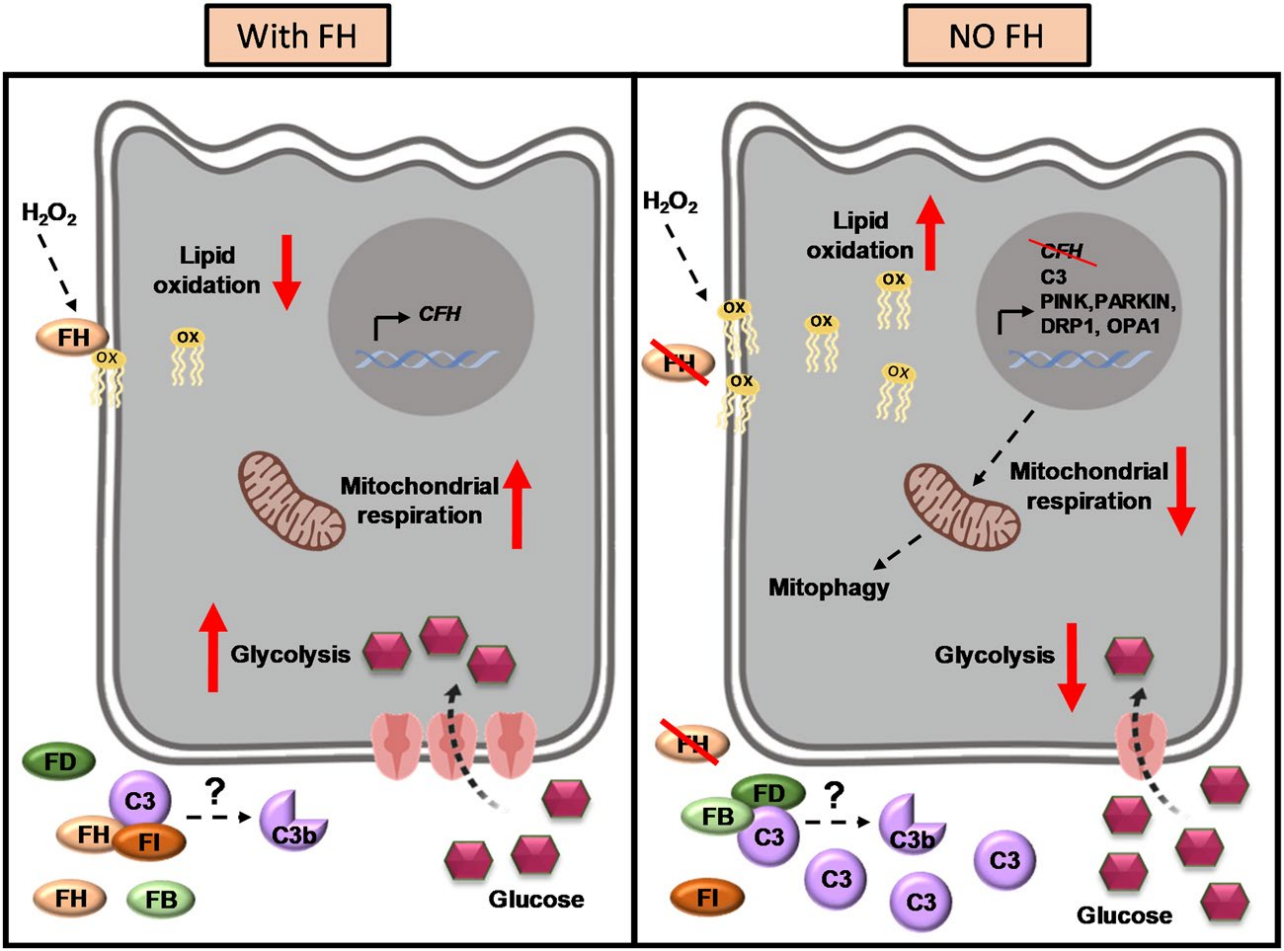
Schematic representation of RPE cell behaviour in presence (left panel) and absence (right panel) of Factor H (FH).

In conclusion, this study provides insight into a new mechanism by which FH dysregulation could contribute to processes relevant to AMD (as summarized in the schema in Fig. 7). FH reduction renders RPE cells more vulnerable to oxidative stress, with the lipids being particularly affected. RPE cells lacking functional FH show a reduced bioenergetics profile, regarding both, glycolysis and oxidative phosphorylation. We hypothesize the involvement of mitophagy and mitochondrial dynamics in the process. Taken together, our results suggest a non-canonical role of FH in AMD and highlight its protective role in RPE cells against oxidative stress and metabolic reprogramming, which could help our understanding of the early stages of the disease. Future therapeutic strategies that systemically target the complement system may consider that simple systemic inhibition of complement activity alone may be insufficient to successfully treat AMD.

## Material and Methods

### Cell culture

Human retinal pigment epithelium (RPE) cell line hTERT-RPE1 was obtained from the American Type Culture Collection (ATCC). Cells were maintained in Dulbecco’s modified Eagle’s medium (DMEM; Gibco, Germany) containing 10% fetal calf serum (FCS; Gibco, Germany), penicillin (100 U/ml), streptomycin (100 µg/ml) in a humidified atmosphere containing 5% CO_2_.

### Experimental settings

Cells were seeded in complete growth medium without phenol red in 6- or 24-well plates depending on the experiment and allowed to attach overnight. siRNA mixture with Viromer Blue reagent was prepared according to the manufacturer (Lipocalyx, Germany) using a mix of 3 different double strand hairpin interference RNAs specific for *CFH* and a negative control (Neg), recommended by the provider (IDT technologies, Belgium). In parallel, a positive fluorescent control was used to monitor transfection efficiency (data not shown). Culture medium was substituted with fresh medium and siRNA mixture was added dropwise. After 24 hours, cells were pre-treated for 90 minutes with medium containing 200 µM H_2_O_2_ or PBS as control. Cells were maintained in serum free medium, unless specified otherwise, for the indicated experiment duration. Optimal H_2_O_2_ concentration was assessed in preliminary experiments in hTERT-RPE1 cells, where cell density was monitored using Crystal Violet staining^76^ (Supplementary Fig. S4). Concentration leading to minimal damage (200 µM) was used for further experiments. Rescue experiments were performed by exogenous application of purified FH. After both silencing and H_2_O_2_ pre-treatment, culture media was substituted with serum free medium containing purified FH protein (CompTech, Texas, USA) at the concentration of 1 µg/ml.

### RNA extraction, cDNA synthesis and quantitative RT-PCR

Cell pellets were collected at the indicated time points and were resolved in 1 ml of TriFAST (PeqLab, Germany), homogenized by inversion and incubated at room temperature for 5 minutes. Then, 200 μl of chloroform was added and the cell pellets vortexed for 15 seconds. Samples were left 10 minutes at room temperature and then centrifuged at 12,000 g for 15 minutes at 4 °C. The aqueous phase was transferred into a new tube and mixed with 500 μl of isopropanol for precipitation. After incubation for 10 minutes in ice, samples were centrifuged at 12,000 g for 15 minutes at 4 °C. Pellets were rinsed twice with EtOH 75%, dried, resuspended in 20 μl of RNase-free water. RNA purity and concentration were measured using Nanodrop. cDNA was synthesized *via* reverse-transcription of 2–5 μg of RNA using M-MLV Reverse Transcriptase (200 U, Promega, Wisconsin, USA), random primers (10 ng/μl, Promega, Wisconsin, USA) and dNTPs (0.5 mM) in a total volume of 20 μl. cDNA was used to analyse differences in gene expression by qRT-PCR employing SensiMix SYBR low-Rox KIT (Bioline, Germany) along with gene specific forward and reverse primers (250 nM) listed in Table 1. PCR protocol includes 40 cycles of: 95 °C (15 seconds), 57 °C (15 seconds) and 72 °C (25 seconds). Relative mRNA expression of each gene of interest (GOI) was quantified by using PRPL0 as the housekeeping control gene.

**Table 1:** List of qPCR primers.

gene name	fwd	rev
ND4	5′- CCT CGT AGT AAC AGC CAT TCT C -3	5´- CTG TGA GTG CGT TCG TAG TT -3´
COX4	5′- TGT TGG CTA CCA GGG TAT TTA G -3	5′- CTT CGC TCT TCA CAA CAC TTT C -3
ATP6	5′- CAC TAA AGG ACG AAC CTG ATC TC -3	5′- GAT AGT TGG GTG GTT GGT GTA A -3
OPA1	5′- GAG GAC AGC TTG AGG GTT ATT C -3	5′- CTG CAG AGC CTC TTC CAT AAA -3
PINK1	5′- GGC TTG GCA AAT GGA AGA AC -3	5′- CTC AGT CCA GCC TCA TCT ACT A -3
PARKIN	5′- CCA CAC TAC GCA GAA GAG AAA -3′	5′- GAG ACT CAT GCC CTC AGT TAT G -3′
DRP1	5′- GAG CTT CTT TGC AGC CTT TG -3′	5′- CCA GAA TTG GAA GGG CTA TGT -3
LDHA	5′- ACC CAG ATT TAG GGA CTG ATA AAG -3′	5′- CCA ATA GCC CAG GAT GTG TAG -3
SLC2A1	5′- GAT GGG AGT GAG ACA GAA GTA AG -3	5′- CAC TGA TGA GAG GTA CGT GTA AG -3′
PPARGC1A	5′- AGA GCG CCG TGT GAT TTA T -3′	5′- CTC CAT CAT CCC GCA GAT TTA -3′
NFE2L2	5′- TGA TTC TGA CTC CGG CAT TT -3′	5′- GCC AAG TAG TGT GTC TCC ATA G -3
PRDX3	5′- AGC CAT CTT GCC TGG ATA AAT A -3′	5′- GTA GTC TCG GGA AAT CTG CTT AG -3′
CAT	5′- CTG GAG CAC AGC ATC CAA TA -3′	5′- TCA TTC AGC ACG TTC ACA TAG A -3′
GPX1	5′- CAT CAG GAG AAC GCC AAG AA -3′	5′- GCA CTT CTC GAA GAG CAT GA -3′
BEST1	5′- CTC AGT GTG GAC ACC TGT ATG -3	5′- CCC AAC TAG ACA AGT CAG GAA G -3
RPE65	5′- GGA CTT GGC TTG AAT CAC TTT G -3′	5′- AAG ATG GGT TCT GAT GGG TAT G -3′
PRPLO	5′- GGA GAA ACT GCT GCC TCA TAT C -3′	5′- CAG CAG CTG GCA CCT TAT T -3′
C3	5′- ACG GCC TTT GTT CTC ATC TC -3′	5′- CAA GGA AGT CTC CTG CTT TAG T -3′
*CFH*	5′- CTG ATC GCA AGA AAG ACC AGT A -3′	5′- TGG TAG CAC TGA ACG GAA TTA G -3′

∆CT = CT(GOI)-CT(housekeeping)

∆∆CT = ∆CT(sample)-∆CT(control)

n-fold expression = 2^−∆∆CT(GOI)^

### Western blot

Cell culture supernatants were collected 48 hours after H_2_O_2_ pre-treatment. Following cell debris removal by centrifugation, supernatants were precipitated using ice-cold acetone. Proteins were re-suspended in NuPAGE™ LDS Sample Buffer containing reducing agent (Invitrogen, California, USA), separated on 8–16% or 4–12% SDS-PAGE gels and transferred on PVFD membranes. Membranes were exposed overnight to the primary antibodies (anti-FH, sc-166608, Santa Cruz, Texas, USA; anti-C3, PA5-21349, Invitrogen, California, USA) and for 1 hour to HRP-conjugated anti-mouse or anti-rabbit secondary antibody (1:2.000, Cell Signaling, Massachusetts, USA). Immunoreactivity was visualized with Pierce™ ECL Western Blotting Substrate (Thermo Fisher Scientific, Massachusetts, USA) and detected with FusionFX instrument (Vilber Lourmat, France).

### C3b ELISA

C3/C3b concentration was evaluated in cell culture supernatants by ELISA assay according to the manufacturer’s instructions (Abcam, UK). Samples were loaded undiluted along with standards and controls in 96 well-plates coated with specific C3b antibody. Absorbance was read at a wavelength of 450 nm immediately after the assay procedure at Spark multimode microplate reader (Tecan, Switzerland). Subtraction readings at 570 nm were taken to correct optical imperfections.

### sC5b-9 ELISA

Formation of the terminal complement complex was evaluated in cell culture supernatants and cell lysates using the MicroVue SC5b-9 Plus EIA kit (Quidel, California, USA). Samples were processed and the assay was performed according to the manufacturer’s instructions. Provided positive controls were loaded in parallel. Absorbance was read at a wavelength of 450 nm immediately after the assay procedure at Spark multimode microplate reader (Tecan, Switzerland). All sample values resulted lower than limit of detection (LOD 3.7 ng/ml) and limit of quantification (LLOQ 8.8 ng/ml).

### Cytotoxicity and viability assay

Cytotoxicity and viability were assessed using the ApoTox-Glo™ Triplex Assay (Promega, Wisconsin, USA) according to the manufacturer’s instructions. Briefly, two fluorogenic dyes were added to the cell culture media. Viability was assessed by cell-permeable GF-AFC (glycyl-phenylalanyl-aminofluorocoumarin) dye, which is cleaved by live-cell proteases and fluorescence signal is read at 400_Ex_/505_Em_. Cytotoxicity, defined by cell membrane damage, was assessed by cell-impermeable bis-AAF-R110 (bis-alanylalanyl-phenylalanyl-rhodamine 110) dye, which is cleaved by dead-cell proteases released in the cell culture supernatants after membranes damages. Fluorescence is read at 485_Ex_/520_Em_. Spark multimode microplate reader (Tecan, Switzerland) was used for fluorescence measurements. The cleaved products have different excitation/emission readouts; therefore, simultaneous measurements of viability and cytotoxicity were possible. Data are normalized to untreated siNeg controls.

### Lipid peroxidation detection

Lipid peroxidation in live cells was measured *via* Image-iT® Lipid Peroxidation Kit (Thermo Fisher Scientific, Massachusetts, USA), based on BODIPY® 581⁄591 C11 fluorescent dye. At the indicated time point, the dye was added to cell culture media at a final concentration of 5 µM. Following incubation and washing steps, fluorescence was measured at Spark multimode microplate reader (Tecan, Switzerland). Upon oxidation, the reagent shifts the fluorescence emission peak from ~590 nm to ~510 nm. Data are shown as ratio of oxidized/reduced lipids.

### Mitochondrial respiration

Mitochondrial function was assessed in live cells using an XFp Extracellular Flux Analyzer (Agilent Technologies, California, USA). After 24 hours silencing (siNeg vs si*CFH*) in 6-well-plates, hTERT-RPE1 cells (3 × 10^4^ cells/well) were seeded in at least duplicates in XFpSeahorse microplates and allowed to adhere overnight. Cells were pre-treated for 90 minutes with medium containing 200 µM H_2_O_2_ or PBS. Following medium change, cells were grown for further 48 hours. Measurements of oxygen consumption rate (OCR) were performed in freshly prepared assay medium, pH 7.4 (Cell Mito Stress Test Assay Medium), according to the manufacturer’s protocol. OCR was measured before and after the serial addition of inhibitors to assess several parameters of mitochondrial function (shown in Fig. 5a). First, 10 μΜ Oligomycin, a complex V inhibitor, was injected directly after the basal measurements, inhibiting ATP synthase and resulting to a reduction in OCR. This decrease in OCR is linked to cellular ATP production. Next, 10 μΜ FCCP, an uncoupling agent, was injected to collapse the proton gradient and disrupt the mitochondrial membrane potential, bringing oxygen consumption rate to maximal levels. The difference between maximal respiration and basal respiration is defined as reserve respiratory capacity. Lastly, 2 μΜ Antimycin A and 1 mΜ Rotenone, inhibitors of complex III and I, respectively, were added to shut down the mitochondrial respiration and enable the calculation of non-mitochondrial respiration. Following each injection, 3 measurements for a total period of 15 minutes were recorded. The data were analyzed using Wave 2.6 Software. Cell number was evaluated after the experiment, using Crystal Violet assay, and no difference was observed among different wells, therefore the cell number was not taken into account for the normalization. At least 2 technical replicates per condition were used and all measurements from 4–8 independent experiments were normalized to the mean of the baseline values of siNeg controls. All reagents were purchased from Sigma-Aldrich; Missouri, USA.

### Glycolysis

Glycolysis function was assessed in live cells using an XFp Extracellular Flux Analyzer (Agilent Technologies, California, USA). After 24 hours silencing (siNeg vs si*CFH*) in 6-well-plates, hTERT-RPE1 cells (3 × 10^4^ cells/well) were seeded in at least duplicates in XFpSeahorse microplates and allowed to adhere overnight. Cells were pre-treated for 90 minutes with medium containing 200 µM H_2_O_2_ or PBS. Following medium change, cells were grown for further 48 hours. Measurements of extra-cellular acidification rate (ECAR) were performed in freshly prepared assay medium, pH 7.4 (Glycolysis Stress Test Assay Medium), according to the manufacturer’s protocol. Assay procedures are reported in Fig. 3a. The basal levels of ECAR were measured in medium without glucose or pyruvate. Then, 10 mM Glucose was injected activating the glycolytic pathway towards pyruvate production and causing a rapid increase in ECAR, which is reported as the basal glycolytic rate. Then, 10 μM Oligomycin, an ATP synthase inhibitor was injected, in order to inhibit mitochondrial ATP production, and shift the energy production towards glycolysis, revealing the maximal glycolytic capacity of the cells. The difference between maximal glycolytic capacity and basal glycolysis defines the glycolytic reserve. Lastly, 50 mM 2-deoxy-glucose (2-DG), a glucose analog that inhibits glycolysis was injected, resulting in ECAR decrease. Following each injection, 3 measurements for a total period of 15 minutes were recorded. The data were analyzed using Wave 2.6 Software. Cell number was evaluated after the experiment, using Crystal Violet assay, and no difference was observed among different wells, therefore the cell number was not taken into account for the normalization. At least 2 technical replicates per condition were used and all measurements from 4–8 independent experiments were normalized to the mean of the baseline values of siNeg controls. All reagents were purchased from Sigma-Aldrich; Missouri, USA.

### Glucose uptake

Glucose uptake was assessed using Glucose Uptake-Glo Assay (Promega, Wisconsin, USA) according to the manufacturer’s instructions. Briefly, at the desired time point cells were washed with PBS and incubated 10 minutes with 1 mM glucose analogue 2-deoxy-glucose (2DG), which can be phosphorylated into 2-deoxy-glucose-6-phosphate (2DG6P), but not be processed further by the glycolysis enzymes. After addition of Stop Buffer and neutralization Buffer, 2DG6P Detection reagent containing glucose-6-phosphate dehydrogenase (G6PDH), NADP+, reductase, Glo-luciferase and luciferin substrate was added to allow detection. Luminescence was recorded using Spark multimode microplate reader (Tecan, Switzerland).

### Immunohistochemistry

hTERT-RPE1 cells were grown on coverslips, fixed for 20 minutes at room temperature (RT) in 4% paraformaldehyde in PBS, permeabilized for 5 minutes at RT in 0.3% Triton X-100 in PBS and saturated for 1 hour RT in normal goat serum 10%, BSA 1% in PBS. Cells were stained overnight at 4 °C with anti-ZO-1 primary antibody (610966, BD Transduction Laboratories, California, USA). After washes, cells were incubated for 1 hour at RT with anti-mouse secondary antibody conjugated to Alexa Fluor 488 (A-11029, Thermo Fisher Scientific, Massachusetts, USA). Cells were stained with DAPI for 5 minutes and coverslips were mounted with Fluoromount-G solution (17984-25, Electron Microscopy Sciences, Pennsylvania, USA). Images were obtained using Z-stacks on a Zeiss Axio Imager Z1 ApoTome Microscope. Length of ZO-1 labeled fragments was measured with an ImageJ adjusted customized macro from Borras *et al*.^21^. Enhancement of linear structures was obtained by computation of the smallest eigenvalue of Hessian tensor with the ImageJ plugin FeatureJ ((Erik Meijering, Erasmus University Medical Center, Rotterdam, Netherlands. Plugin available from https://imagescience.org/meijering/software/featurej). Obtained images were binarized and skeletonized. Skeleton were analyzed and the average branch length distribution was compared between siNeg and siCFH.

### Statistical analysis

All data sets were tested for normal distribution. The combined effects of FH reduction and peroxide pre-treatment on lipid peroxidation, viability, cytotoxicity, glucose uptake and gene expression were assessed with two-way analyses of variance (ANOVA). An unpaired Student’s t-test was used to compare data from control cells (siNeg) versus si*CFH* cells and H_2_O_2_-treated cells, as well as to compare siNeg and si*CFH* cells after pre-treatment with H_2_O_2_. ELISA data were analyzed using a paired Student’s t-test. Bioenergetics data were subjected to outliers’ identification *via* ROUT method. An unpaired Student’s t-test (or Mann-Whitney test in case the dataset did not pass a normality test) was used to compare data from control cells (siNeg) versus si*CFH* cells and H_2_O_2_-treated cells, as well as to compare siNeg and si*CFH* cells after pre-treatment with H_2_O_2_. Combined effects of FH reduction and peroxide pre-treatment on metabolic parameters were analyzed using two-way ANOVA. Analyses were performed using GraphPad Prism 8 software. Western Blot images were analyzed for signal quantification using Fiji (ImageJ). Significance level was set at p < 0.05.

## Supplementary information

Supplementary Information.
