# Supplementary material for: Loss of Complement Factor H impairs antioxidant capacity and energy metabolism of human RPE cells
Source: Sci Rep. 2020 Jun 25;10:10320. doi: 10.1038/s41598-020-67292-z (PMC7316856; doi:10.1038/s41598-020-67292-z)
Supplement: Supplementary file 1 — Supplementary Information. [file 41598_2020_67292_MOESM1_ESM.pdf]

Loss of Complement Factor H impairs antioxidant capacity and energy metabolism of human RPE cells

Angela Armento[1], Sabina Honisch[1], Vasiliki Panagiotakopoulou[2, 3], Inga Sonntag[1], Anke Jacob[1], Sylvia Bolz[1], Ellen Kilger[1], Michela Deleidi[2, 3], Simon Clark[1]; Marius Ueffing[1*]

[1]Institute for Ophthalmic Research, Department for Ophthalmology, Tübingen, Germany.

[2]German Center for Neurodegenerative Diseases (DZNE), Tübingen, Germany.
[3]Hertie-Institute for Clinical Brain Research, University of Tübingen, Tübingen, Germany

*corresponding author

Marius.ueffing@uni-tuebingen.de

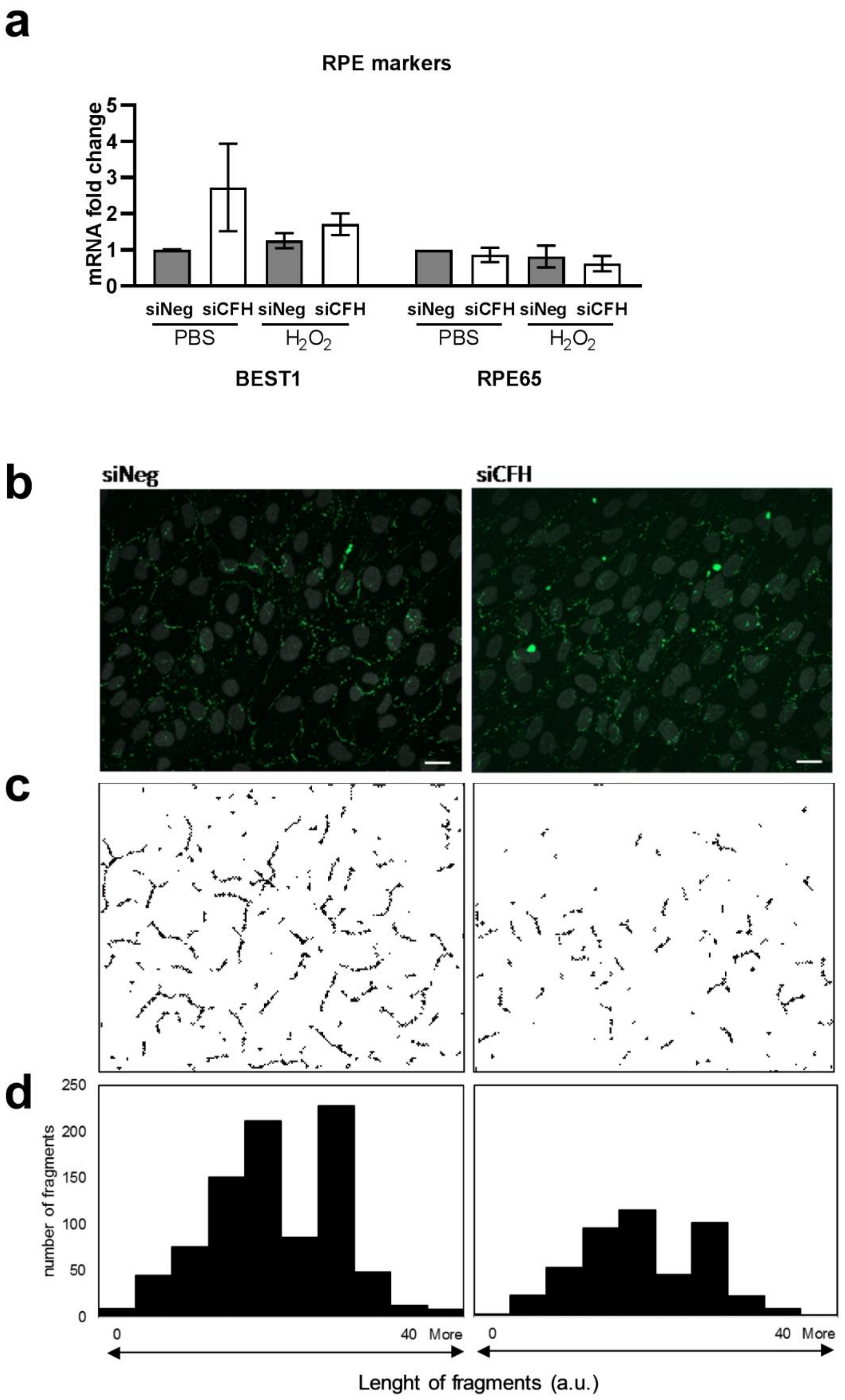

Suppl. Fig. S1. Expression and localization of RPE markers in experimental conditions. Cells were seeded, let attach overnight and silenced for 24 hours with negative control (siNeg) or *CFH* specific (si*CFH*) siRNA. Cells were exposed for 90 minutes to 200 µM H_2O_2 or PBS and after 48 hours RNA was collected. **a.** Gene expression analysis by qRT-PCR of RPE markers: Bestrophin 1 (BEST1), Retinoid

Isomerohydrolase (RPE65), SEM is shown, n=3. Data are normalized to housekeeping gene PRPL0 using Δ ΔCt method. **b**. ZO-1 immunostaining in hTERT-RPE1 cells 24 hours after silencing. **c.** Skeletonized ZO-1 staining based on the images in b. **d**. Histograms showing the quantification of the number and length of ZO-1 fragments, n=6. a.u. arbitrary units. Scale bars: 20 µm.

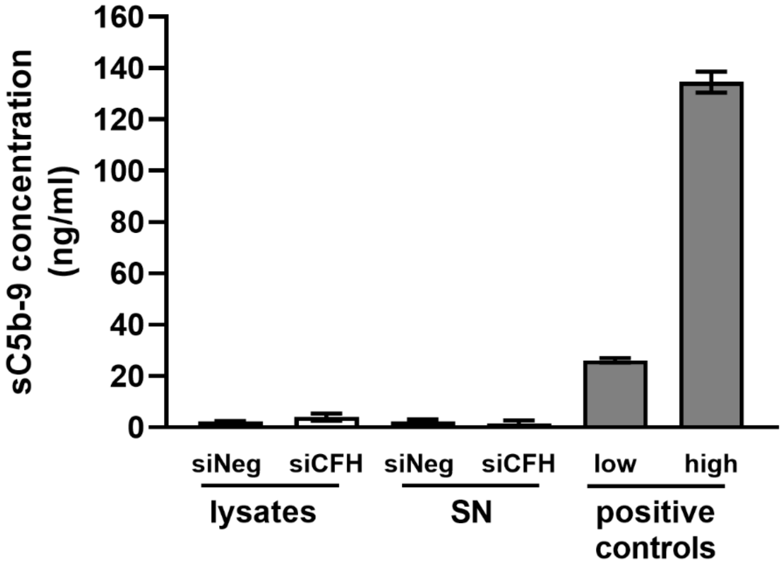

Suppl. Fig. S2. Levels of sC5b-9 in RPE cells. hTERT-RPE1 cells were seeded, let attach overnight and silenced for 24 hours with negative control (siNeg) or *CFH* specific (si*CFH*) siRNA. Cell lysates and cell culture supernatants were collected after 48 hours. sC5b-9 ELISA was performed on lysates and cell culture supernatants (SN) and positive controls provided by the manufacturer. SEM is shown, n=3. Lower limit of detection (LOD) 3.7 ng/ml. Lower limit of quantification (LLOQ) 8.8 ng/ml.

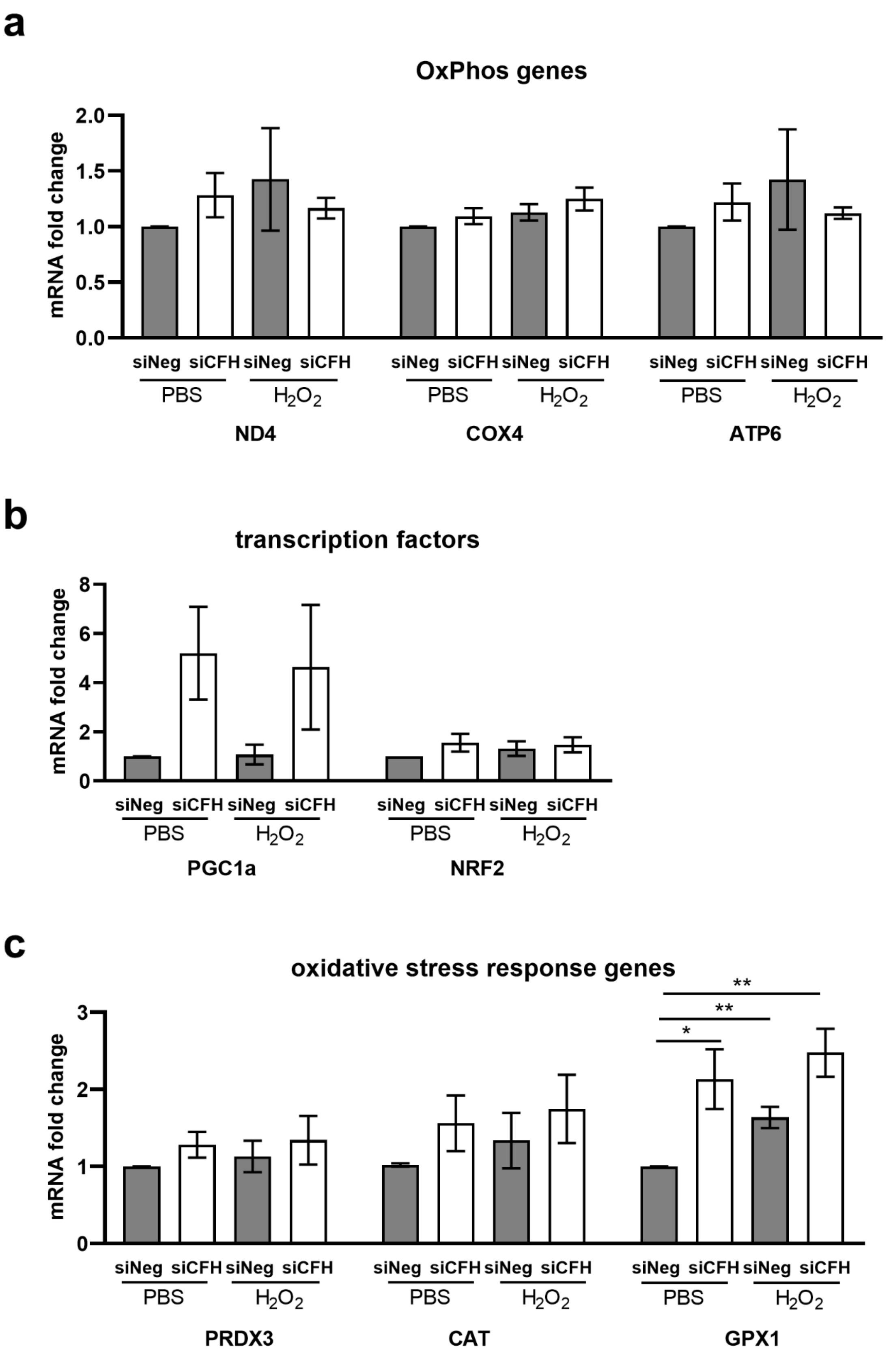
a
OxPhos genes
mRNA fold change
2.0
1.5
1.0
0.5
0.0
siNeg siCFH siNeg siCFH
PBS
H_2O_2
ND4
COX4
ATP6
b
transcription factors
mRNA fold change
8
6
4
2
0
PGC1a
NRF2
c
oxidative stress response genes
mRNA fold change
3
2
1
0
*
**
PRDX3
CAT
GPX1

Suppl. Fig. S3. Expression of OxPhos genes, transcription factors and oxidative response genes in experimental conditions. hTERT-RPE1 cells were seeded, let attach overnight and silenced for 24 hours with negative control (siNeg) or *CFH* specific (si*CFH*) siRNA. Cells were exposed for 90 minutes to 200 µM H_2O_2 or PBS and after 48 hours RNA was collected. **A** Gene expression analysis by qRT-PCR of OxPhos genes: NADH dehydrogenase 4 (ND4), Cytochrome c oxidase subunit 4 (COX4) and mitochondrially encoded ATP synthase 6 (ATP6). SEM is shown, n=3 **B** Gene expression analysis by qRT-PCR of transcription factors: Peroxisome Proliferator-Activated Receptor Gamma Coactivator 1-Alpha (PGC1a/PPARGC1A) and Nuclear Factor, Erythroid 2 Like 2 (NRF2/NFE2L2). SEM is shown, n=3 **C** gene expression analysis by qRT-PCR of genes involved in oxidative stress response: peroxiredoxin 3 (PRDX3), catalase (CAT), Glutathione Peroxidase 1 (GPX1). SEM is shown, n=3. Data are normalized to housekeeping gene PRPL0 using Δ ΔCt method. Significance was assessed by Student t-test (single effect) and two-way ANOVA (combined effects) as described in the methods section. * $p<0.05$, ** $p<0.01$.

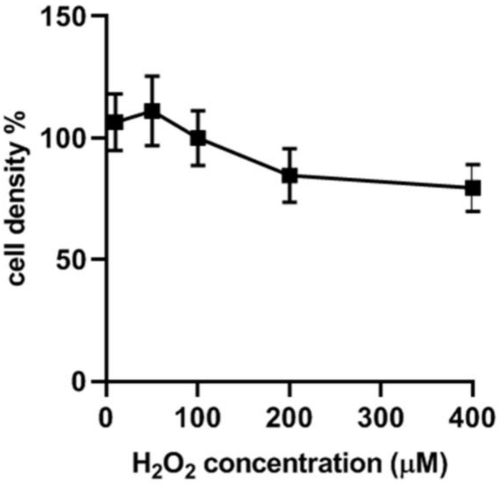

Suppl. Fig. S4. Assessment of optimal concentration for H_2O_2 pre-treatment. hTERT-RPE1 cells were seeded, let attach overnight and exposed for 90 minutes to increasing concentrations of H_2O_2 and after 48 hours cell density was analyzed via Crystal Violet staining.

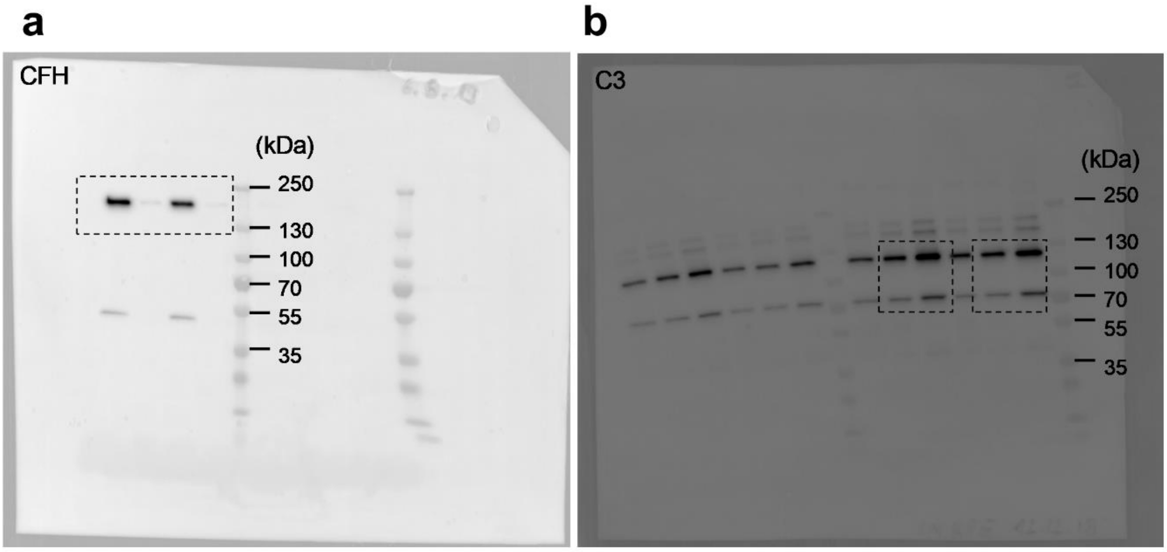

Suppl. Fig. S5. Original full images of Western Blots acquired with FusionFX instrument (Vilber Lourmat, France) and software provided by the manufacturer using authomatic modus. **a**. corresponds to Fig. 1b. **b** corresponds to Fig. 1d. Boxes highlight the cropped bands reported in Fig. 1. Additional bands represent the cleaved forms of the protein of interest.

The EYE-RISK Consortium

Soufiane Ajana[1], Blanca Arango-Gonzalez[2], Angela Armento[2], Franz Badura[4], Ulrich Bartz-Schmidt[2], Berta De la Cerda[5], Marc Biarnés[6], Anna Borrell[6], Johanna M. Colijn[8,9], Audrey Cougnard-Grégoire[1], , Eiko K. de Jong[10], Cécile Delcourt[1], Anneke I. den Hollander[10,11], Sigrid Diether[2], Eszter Emri[12], Tanja Endermann[3], Lucia L. Ferraro[6], Míriam Garcia[6], Thomas J. Heesterbeek[10], Sabina Honisch[2], A Ikram[8], Eveline Kersten[10], Ellen Kilger[2], Caroline C.W. Klaver[8,9,10], Eloed Kortvely[2,13], Hanno Langen[13], Claire Lastrucci[18], Imre Lengyel[12], Phil Luthert[14], Magda Meester-Smoor[8,9], Bénédicte M.J. Merle[1], Jordi Monés[6], Everson Nogoceke[13], Tunde Peto[15], Frances M. Pool[16], Eduardo Rodríguez[6], Luis Serrano [18], Marius Ueffing[2], Timo Verzijden[8,9], Johannes Vingerling[9], Markus Zumbansen[17].

[1] Univ. Bordeaux, Inserm, Bordeaux Population Health Research Center, team LEHA, UMR 1219, Bordeaux, France. [2] Department of Ophthalmology, Institute for Ophthalmic Research, Eberhard Karls University Tuebingen, University Clinic Tuebingen, Tuebingen, Germany. [3] Assay Development, AYOXXA Biosystems GmbH, Cologne, Germany. [4] Pro-Retina Deutschland, Aachen, Germany. [5]Department of Regeneration and Cell Therapy, Andalusian Molecular Biology and Regenerative Medicine Centre (CABIMER), Seville, Spain. [6] Barcelona Macula Foundation, Barcelona, Spain. [7] Business Development, AYOXXA Biosystems GmbH, Cologne, Germany. [8] Department of Epidemiology, Erasmus Medical Center, Rotterdam, the Netherlands. [9] Department of Ophthalmology, Erasmus Medical Center, Rotterdam, the Netherlands. [10] Department of Ophthalmology, Radboud University Medical Center, Nijmegen, the Netherlands. [11] Department of Human Genetics, Radboud University Medical Center, Nijmegen, the Netherlands. [12] Centre for Experimental Medicine, Queen's University Belfast, Belfast, United Kingdom. [13] Roche Innovation Center Basel, F. Hoffmann-La Roche Ltd, Basel, Switzerland. [14] Institute of Ophthalmology, University College London, London, United Kingdom. [15] Centre for Public Health, Queen's University Belfast, Belfast, United Kingdom. [16] Ocular biology, UCL Institute of Ophthalmology, London, United Kingdom. [17]Research and & Development, AYOXXA Biosystems GmbH, Cologne, Germany. [18] Centre for Genomic Regulation (CRG), Barcelona Institute of Science and Technology, Barcelona, Spain.
